# Are 6-month-old human infants able to transfer emotional information (happy or angry) from voices to faces? An eye-tracking study

**DOI:** 10.1371/journal.pone.0194579

**Published:** 2018-04-11

**Authors:** Amaya Palama, Jennifer Malsert, Edouard Gentaz

**Affiliations:** 1 SensoriMotor, Affective and Social Development Laboratory, Faculty of Psychology and Educational Sciences, Universty of Geneva, Geneva, Switzerland; 2 Swiss Center for Affective Sciences, Campus Biotech, University of Geneva, Geneva, Switzerland; 3 CNRS, Grenoble, France; Swinburne University of Technology, AUSTRALIA

## Abstract

The present study examined whether 6-month-old infants could transfer amodal information (i.e. independently of sensory modalities) from emotional voices to emotional faces. Thus, sequences of *successive* emotional stimuli (voice or face from one sensory modality -auditory- to another sensory modality -visual-), corresponding to a cross-modal transfer, were displayed to 24 infants. Each sequence presented an emotional (angry or happy) or neutral voice, uniquely, followed by the simultaneous presentation of two static emotional faces (angry or happy, congruous or incongruous with the emotional voice). Eye movements in response to the visual stimuli were recorded with an eye-tracker. First, results suggested no difference in infants’ looking time to happy or angry face after listening to the neutral voice or the angry voice. Nevertheless, after listening to the happy voice, infants looked longer at the incongruent angry face (the mouth area in particular) than the congruent happy face. These results revealed that a cross-modal transfer (from auditory to visual modalities) is possible for 6-month-old infants only after the presentation of a happy voice, suggesting that they recognize this emotion amodally.

## Introduction

Expressing emotions via facial expressions, voices or even body movements helps to transmit one’s internal state and intentions to others [1]. Human infants are able to recognize emotions expressed by the people in their environment (parents, brothers and sisters, etc.), this adaptive ability is essential for infants to interact with these people [2]. However, perceiving emotional expressions is not trivial for infants and the development of this ability depends on the type of emotions expressed and their mode of presentation [3–5].

The spontaneous visual preference for happy faces, observed in specific conditions in newborns [6,7] generally persists until 5 months of age and seems to decline after that. More particularly, at 3 months old, the amount of time infants look at a happy face is greater than the amount of time they spend looking at a neutral one [8]. Additionally, at 4 months old, infants’ first fixations are more often directed toward happy faces than neutral faces [9]. Nevertheless, results show that this visual preference is influenced by other facial dimensions, for example, the preference for happy faces is limited to female faces in 3.5-month-olds [10]. This difference may be explained by the different experiences with male and female faces acquired over the first few days of life [11]. Although this preference for happiness is not reported after 5 months [12], it may still be observed in some cases at 7 months [13,14].

The visual discrimination between happiness and other expressions is demonstrated from 2 to 5 months [3]. Discrimination between surprised [15,16] and angry (frowning) faces [17] occurs at 3 months, and between happy and sad faces at 3–5 months [18]. At 5 months, infants are able to discriminate between happy and neutral faces [19], as well as between happy and fearful faces [20]. Studies have shown a categorical discrimination between happiness and several other emotions (surprise, sadness, fear) for 6–7 month-old children (for reviews [3–5]), as demonstrated by identity-invariant categorization (i.e. infants can categorize the emotion presented by a different identity as the same emotion) e.g. [5,15,18,21,22] for audio-visual stimuli and categorical boundary effects (i.e. in emotional morphing, the point in the continuum of emotional expression when the infant perceived the face as a specific emotional category) (e.g. [23]). However, no evidence for a valence-based categorization of expressions (i.e. the categorization between the same emotional valence, positive or negative) was found in 7-month-old infants [24]. Overall, the positive discrimination for specific contrasts appears earlier in paradigms involving just one or a limited number of face identities (e.g. [17]), and later in paradigms involving the extraction of expressions across multiple identities [15].

A review of the studies regarding the perceptual development of emotional expressions suggests that the sensory mode in which a stimulus is presented, whether it be unimodal or multimodal, plays a significant role in an infant’s ability to discriminate emotions [3]. For example, Flom and Bahrick [25] showed that infants can discriminate among happiness, anger and sadness as of 5 months with unimodal auditory stimuli and as of 7 months with unimodal visual stimuli. Furthermore, at 4 months, infants are able to discriminate among happiness, anger and sadness with multimodal dynamic (audio–visual) stimuli, i.e. when the sounds and the emotional faces are shown simultaneously and synchronized. More evidence of multimodal matching has been reported in 3–4 month-old infants for audio-visual matching of happiness versus sadness (concordant > discordant) and happiness versus anger (discordant > concordant) expressions of the mother [26], as well as for visual-olfactory matching of happy versus disgusted expressions [27]. However, the visual-olfactory matching appears limited to the happy expression and is not present at 5 months. Evidence of audio-visual matching also exists for happy and angry (concordant > discordant) expressions at 6–7 months [13,28].

It should be noted that most of the previous behavioral studies used videos in individual testing sessions. Generally, the experimenters manually coded the infant’s gaze as being either to the left side, the right side, or outside of the screen—generating raw-looking data. This analysis procedure is not very accurate and does not allow for an examination of the specific face areas (eyes and mouth) explored by each infant in function of the conditions. For the aim of this study, we recorded the eye movements that occurred in response to the visual stimuli in each of the 6 test phases using an eye-tracker. There have been few studies that have examined ocular movement with an eye-tracking device in infants. However, eye-tracking, which precisely calculates the time and direction of the gaze, allows for spatial and temporal precision and accuracy. Besides fixation and saccades, the eye-tracker allows one to examine specific areas of interest (AOIs) on the stimulus presented, such as the eyes and mouth. Depending on the type of emotion, some regions of the face may be more useful than others in helping an infant to determine an emotion. Schurgin et al.’s study [29] shows that by observing an adult’s eye-movements on a picture, one can predict the emotion that is presented on it.

A recent study [30] using eye-tracking and dynamic emotional faces with infants aged from 3 to 12 months, showed that younger infants focused their attention on the eyes and the external features of emotional faces. However, the visual attention of older infants (7- and 12-month-olds) depended on the emotion that was displayed. In this study, the mouth drew the most attention for smiling faces, the eyes and eyebrows drew the most attention for fearful and angry faces, and the upper nose area drew attention for disgusted faces. Another study by the same authors [31] demonstrated that 7-month-old infants looked longer at areas of interest of a neutral face according to the valence of the odors smelt before. With a pleasant (strawberry) scent, the infants looked more at the neutral face, particularly the eyes, eyebrows, nose and mouth areas, whereas with an unpleasant (strong cheese) odor, they looked more at the upper nose area. As a function of the internal states provoked by the smell, the infants searched for reaction cues on the faces presented. Amso et al. [32] found a positive correlation between the time spent looking at the eye area and the ability to discriminate between happy and fearful expressions after having been habituated to fearful expressions at 6 to 11 months old. In another study, Hunnius et al. [33] showed that 4- and 7-month-old infants looked less at inner feature area (mouth, eye and nose) of threat-related expressions (anger or fear) compared to non-threat-related expressions (happy, sad or neutral).

Nonetheless, the existence of the ability to discriminate emotional expressions in unimodal or multimodal conditions does not allow us to determine whether it results from an amodal representation of the emotion or from a sensitivity to specific perceptual features, whether they be visual and/or auditory. Some studies showed that infants use cues such as the salience of teeth at 4 months, rather than emotions, when comparing two emotional faces [34]. The findings of the behavioral studies which used experimental paradigms involving just one or a limited number of face identities do not prove that infants are unable to form emotional representations. However, they confirm that sensitivity to perceptual variables contributes to infants’ performances in many experiments designed to assess sensitivity to emotion.

A relevant way to rule out this difficulty is to study the recognition of emotional expression in a cross-modal task (for review [35]). These data gave evidence that infants can code information in an auditory or tactile mode and then perceive this information in a visual mode, despite several differences in size, volume, texture, shape, etc (such as number [36] or object unity [37]). In this perspective, a similar way to address the question of amodal representation of emotions would be to investigate cross-modal emotional correspondence from auditory to visual emotional stimuli.

The aim of this present study is to evaluate if the ability to discriminate emotional expressions is founded on the nature of emotion *per se*, amodally (i.e. independently of sensory modalities) or on specific physical characteristics of stimuli (faces or voices). To help answer this question, we chose to use a paradigm with a successive cross-modal transfer from emotional voices to emotional faces. To our knowledge, no such experiment has been conducted on infants. This cross-modal transfer of emotional information from auditory to visual consists of two successive phases: an auditory familiarization phase and then a visual test phase. This task is very difficult because it involves a serial mapping process in which emotional information is extracted in an audio format and transformed into a visual format. Thus, if infants are able to successfully transfer the emotional information, it would support the hypothesis that they are able to recognize the emotions amodally, not simply via physical features (pictorial or acoustic). The studies showing the categorical discrimination of emotions (i.e. the extraction of expressions across multiple identities) also support the hypothesis that infants are able to form amodal emotional representations.

Our experiment consists of six sequences of cross-modal transfers that were individually shown to each infant. This study began with a baseline condition in which a neutral voice was presented for 20 seconds during 2 trials, followed by the two emotional faces (happy and angry) presented simultaneously for 10 seconds. The goal was to obtain the baseline of any spontaneous preferences of the looking time between happy and angry faces. This continued with the experimental conditions in which infants received four different sequences corresponding to an emotional voice (happy or angry) presented for 20 seconds (auditory familiarization phase), followed by the two emotional faces being presented simultaneously (one familiar and the other novel vis-à-vis the emotional voice) for 10 seconds (visual test phase without any sound).

We hypothesized that if infants had an amodal representation of emotion, they would be able to detect the correspondence between an emotional voice and a visual face containing the same emotion. In this case, a reaction to novelty was expected: i.e. a longer looking time for the non-matching face. Thus, we expected that infants would prefer the novel face. Furthermore, due to the fact that happiness is the first emotion infants are able to discriminate, we expected the happy expression to be better transferred than the angry one.

Additionally, we examined whether visual preference is dependent on specific areas, such as the eyes and/or mouth of each of the faces, after the auditory familiarization. Interestingly enough, the results from two different infant studies regarding the face areas looked at in function of the emotion presented provided contradictory results. One study [30] showed that infants looked longer at the mouth area for happy faces and at the eyes for angry faces. However, the other study [33] showed that infants look longer at the mouth for the angry faces. Evidently, infants seem to be drawn to these two areas when presented with these emotional faces. Therefore, we examined the mouth and eye areas for both of the emotional faces. In addition, to explore the gaze further, we also examined the first fixations of each visual test phase for each infant and peak looks at the face, the mouth and the eyes for each emotional face.

Finally, we decided to investigate the rarely analyzed gender effect due to the fact that contradictory effects have been reported in previous experiments. Of those that studied this effect, two did not observe any differences between males and females [38, 39] while one observe a significant difference between females and males in emotion recognition, demonstrating that 5-month-old girls recognized emotions similarly to 6-month-old boys [15].

## Method

### Participants

Twenty-four full-term (at least 37 weeks of gestation) 6-month-old infants (13 females; mean age = 6.03 months ± 0.32, range = 6.5–5.2 months) were included in the final sample of the study. Because of the difficulty to apply the eye-tracking technic to infants, a great number of data has been not recorded. Thirty-one additional infants were observed but excluded from the final sample due to technical failure of the eye-tracking not being able to find the pupil (seventeen), excessive movement (two) resulting in loss of gaze data, noisy eye tracking data due to unsuccessful calibration (three) defined as more than 2° of deviation in the x and y axes, inattentiveness to stimuli (looking at the screen less than a third of the entire time) (one), crying (four) or fussiness (four). The descriptive characteristics of the final sample are as follows: the mean age of the mothers was 33.01 (± 4.6) years and 35.56 (± 5.9) years for the fathers. The majority of the parents that participated in the study were married (N = 14) or cohabitating (N = 9), while one parent was a single mother raising her child alone (N = 1). The familys’ socioeconomic status (SES) was calculated using the Largo scale based on paternal occupation and maternal education, ranging from 2 (the highest SES) to 12 (the lowest SES) [40]. The mean socioeconomic status (SES) of the families used in the sample was 3.42 ± 1.47, range = 2–8. Approval for the study was given by the Ethics Committees of the Faculty of Psychology and Educational Sciences of Geneva and all parents gave written informed consent for their children’s participation in the experiment. The experiment was performed in accordance with the relevant guidelines and regulations.

### Stimuli

The emotional nonverbal auditory stimuli of happiness, anger and neutral come from the "Montreal Affective Voice" database [41]. They are expressive onomatopoeic stimuli based on the emission of the vowel /a/. This auditory stimulus was a loop of a one second voice with a break of 1 second between each repetition for a total clip of 20 seconds. Note that these are the vocal productions of only one woman (ref: SF60). The volume of auditory stimuli presented to babies did not exceed 60 dBA.

**Fig 1:**
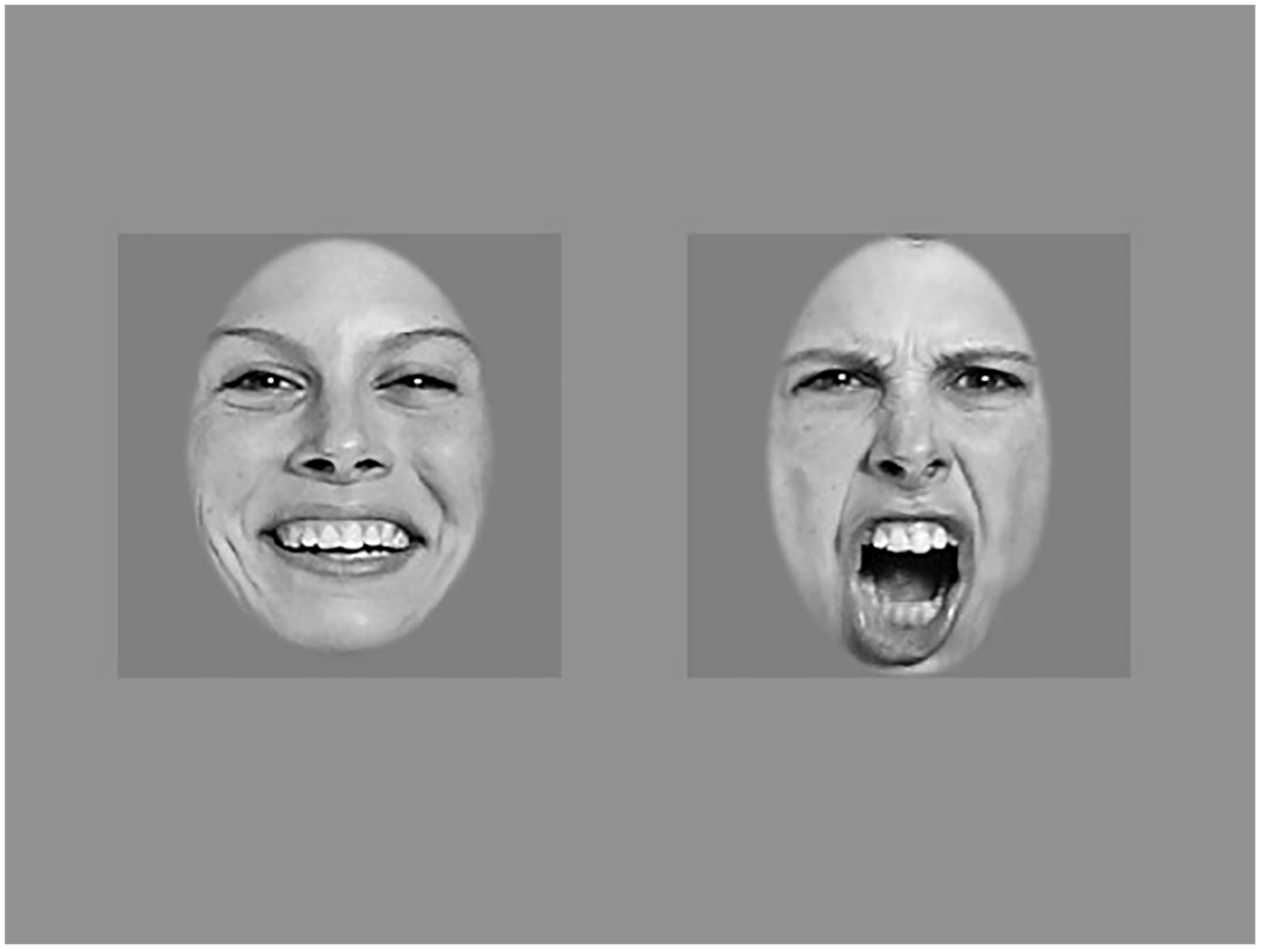
Visual stimuli. The angry face (right) and the happy face (left) with faces from The Karolinska Directed Emotional Faces—KDEF.

The visual stimuli used were emotional (happy and angry) faces of a woman taken from the database "The Karolinska Directed Emotional Faces—KDEF" [42]. These pictures are 9.1 x 9.1 cm, in black and white, and are presented on a medium gray background (RGB 100, 100, 100). The hair is not visible on the stimuli to avoid potential biases of attention on the external elements of the face [43]. Because studies showed that 4-month-old infants discriminate female faces more easily than male faces [10], we tested the emotional faces represented by the same woman (ref: SF4). Faces are presented in pairs, pseudo-randomized for the left and right presentation (Fig 1).

### Experimental procedure

Each infant was comfortably installed in a suitable seat, placed in an experimental cubicle in Geneva’s Baby Lab. The stimulus display screen measured 47.5 cm x 30 cm with a spatial resolution of 1680 x 1050 pixels. The baby was placed at a distance of 60 cm from the screen, at this distance, visual stimuli were 8.7° of visual angle. To focus the infant’s attention on the screen, just before starting the experiment, we presented a cartoon extracted from “Le Monde des petits”. The gaze on visual stimuli was recorded with an eye-tracker SMI RED 250 (SensoMotoric Instruments GmbH, Teltow, Germany).

The experiment started with a 5-point calibration phase with the eye-tracker, an animated image at 5 different locations covering the whole surface of the screen. This phase was repeated until a satisfactory calibration (less than 2° of deviation in the x and y axes) was achieved.

In this experiment, each trial consisted of exposure to a voice (neutral, happy or angry prosody) for 20 seconds accompanied by a black display screen, for an auditory familiarization phase. Afterwards, a pair of emotional faces (happy and angry) was presented for 10 seconds during the visual test phase. The side of presentation of the happy and angry faces were counterbalanced for each voice.

**Fig 2:**
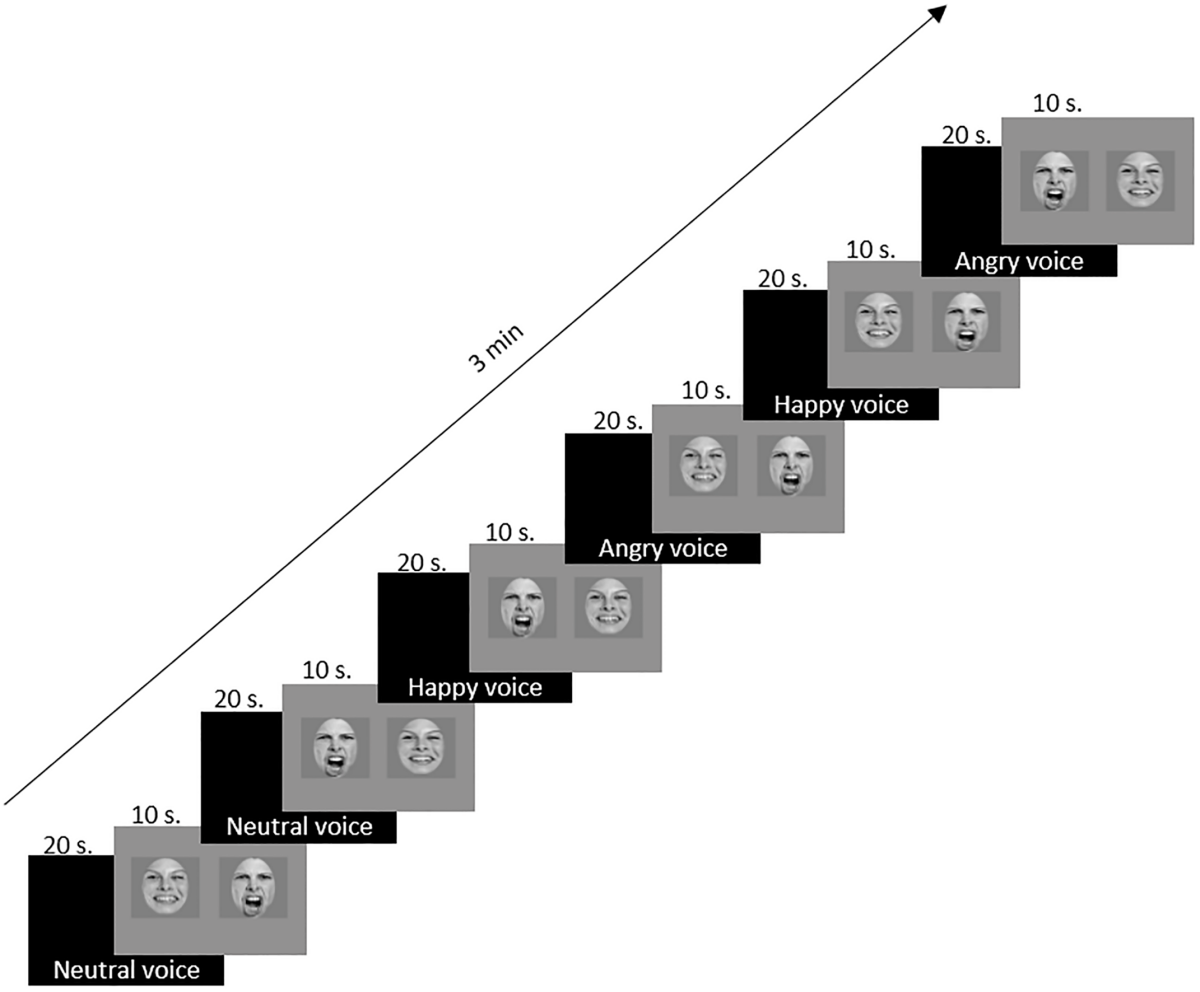
Schematic representation of the successive presentation of all stimuli.

The experiment was composed of 6 trials in this order: first, in order to obtain the baseline of spontaneous preferences for infants, a neutral voice was presented during the first 2 trials, followed by the 2 emotional faces which were laterally counterbalanced. The next 4 trials, the test trials, consisted of the presentation of the 2 emotional voices, each followed by the 2 emotional faces, laterally counterbalanced for each emotional voice, in succession. The happy voice was presented first, to avoid the triggering of a negative reaction by the negative stimulus [44]. The presentation of the 6 trials (sequences of audio-visual transfer) lasted 3 minutes for each infant (Fig 2).

### Data analysis

**Fig 3:**
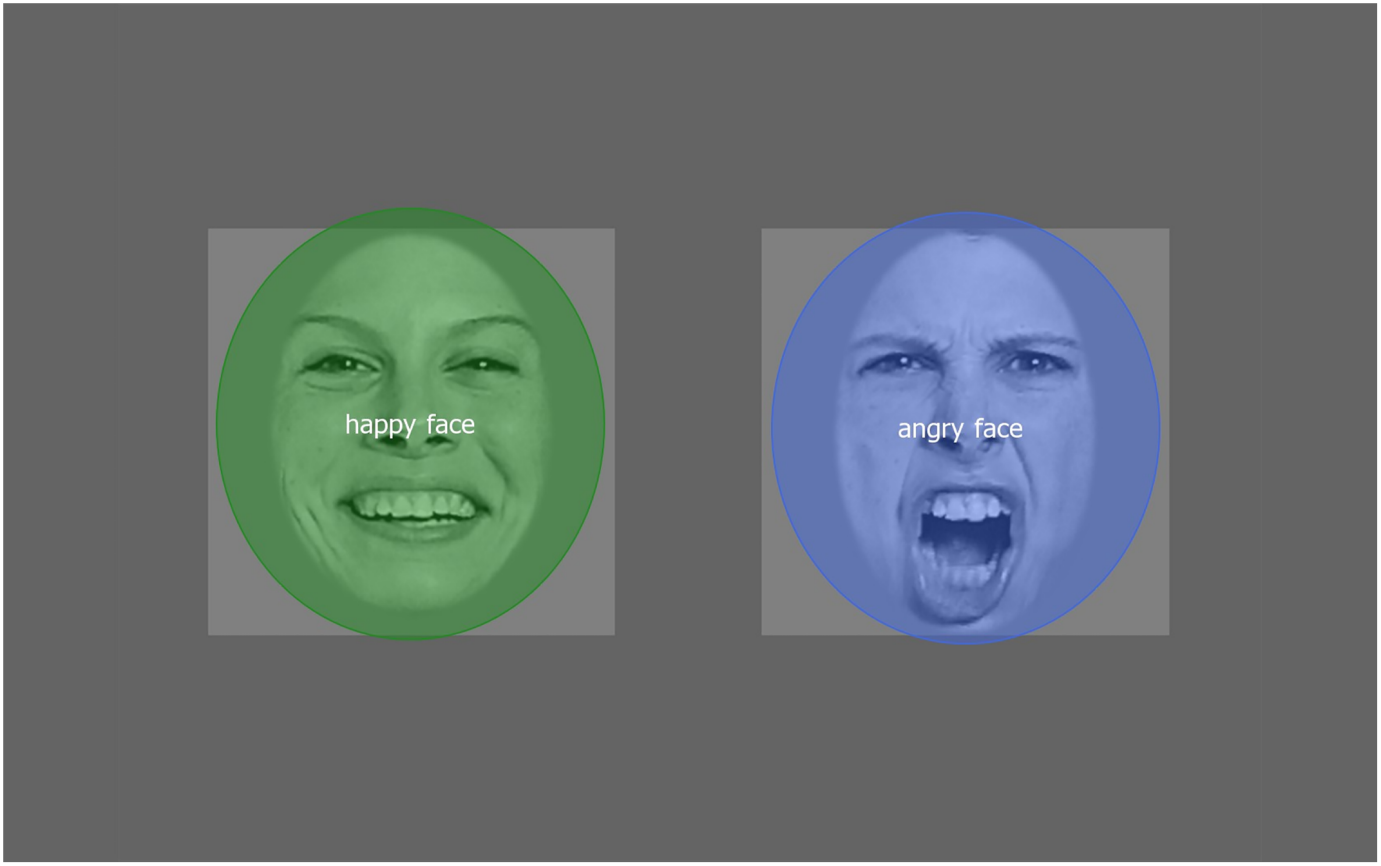
Area of interest representing the whole face. The angry face (right) and the happy face (left). Faces from The Karolinska Directed Emotional Faces—KDEF.

**Fig 4:**
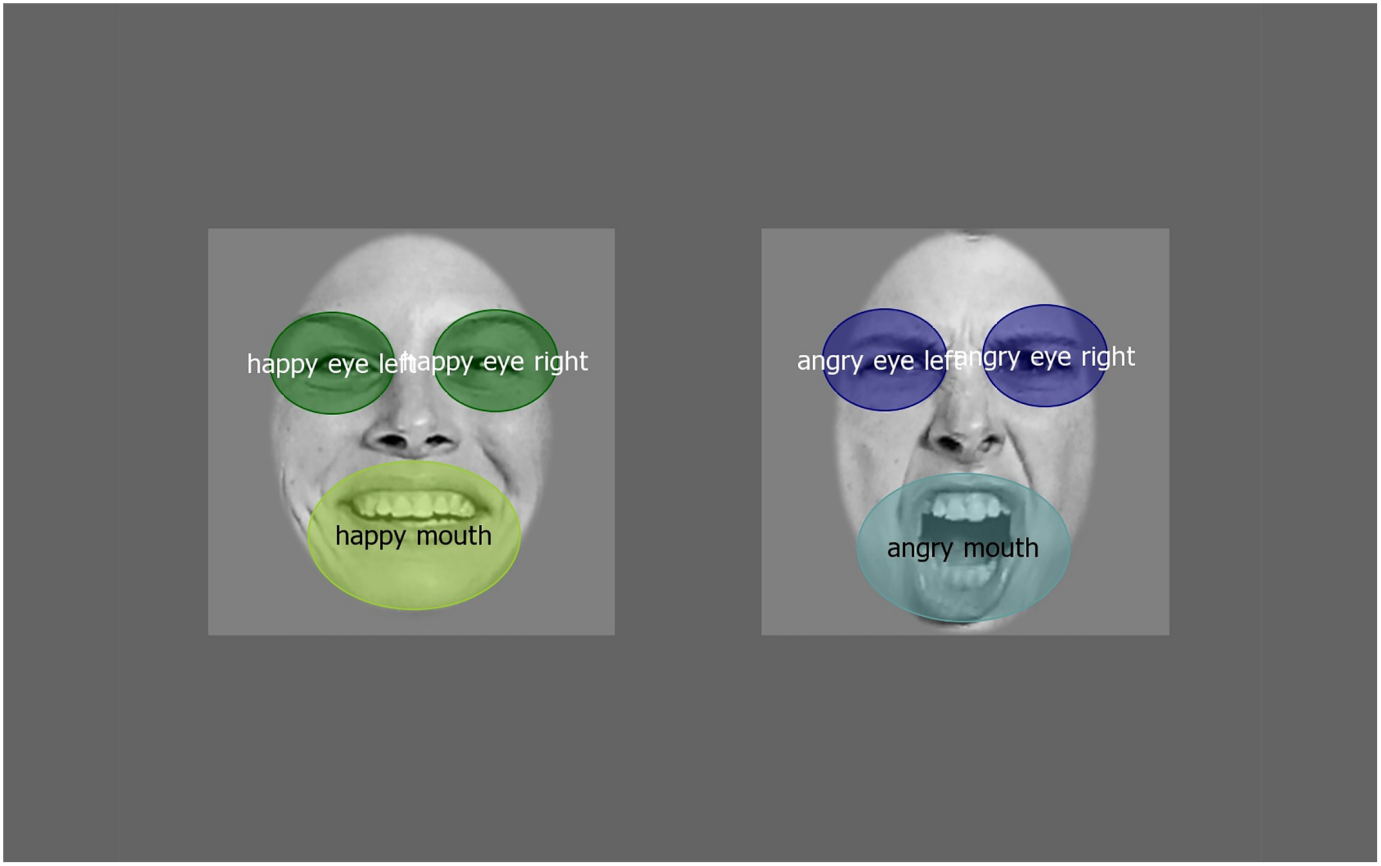
Area of interest representing the eyes and the mouth. The angry face (right) and the happy face (left). Faces from The Karolinska Directed Emotional Faces—KDEF.

All the data were extracted by using Begaze SMI’s analyzer software. The total looking time in seconds to the whole face and to the Areas of Interest (AOI) was calculated by the net dwell time (length of time spent looking the AOIs). We defined AOIs as one general for the whole face (Fig 3) and two specific ones for the eyes and the mouth (Fig 4) for each type of emotional expression (data in S1 Dataset). Peak look duration was calculated in milliseconds as the longest unbroken look at the screen for the same 3 AOIs in each emotional face (data in S2 Dataset). We performed repeated measures analysis of variance (ANOVA) on the whole face and specific AOI looking times and peak looks. The proportion of first fixations toward the faces of each trial (24 infants x 2 trials by voice = maximum 48 first looks for each voice) were also analyzed with T-test (data in S3 Dataset). Statistical analyses were conducted using Statistica 13. The significance threshold was .05 and Bonferroni test was performed to determine significant differences, effect sizes are given in partial eta-squared *η* for ANOVAs.

## Results

### Baseline condition

Table 1 presents the results of the baseline condition for the looking time at the whole face and AOIs (mouth and eyes) as well as the first fixations for the happy or angry face presented after the neutral voice. We found no significant difference concerning the looking time at the emotional faces *F*(1, 23) = 3.135, *p* = .09, η = 0.12, the first fixations at faces (t(47) = 0.58, p = .56; single Student’s T-test) and the peak looks (*F*(1, 23) = 2.02, *p* = .168, η = 0.08). No difference concerning the looking time at the emotional AOIs of the mouth *F*(1, 23) = 2.89, *p* = .103 or eyes *F*(1, 23) = 0.15, *p* = .701. However, with the peak looks, we found an emotional face effect (*F*(1, 23) = 5.34, *p* < .05, η = 0.18) suggesting angry AOIs triggered a longer fixation (420 ± 67 ms) than the happy ones (307 ± 39). Even more, we found a significant interaction between emotional faces and AOIs (*F*(1, 23) = 5.59, *p* < .05, η = 0.19). A pre-planned comparison showed that the angry mouth (523 ± 125 ms) seemed to involve longer fixations than the happy mouth (277 ± 61 ms) (*F*(1, 23) = 6.93, *p* < .05) while both the angry and happy eyes were looked at equally (*F*(1, 23) = 0.16, *p* = .688. These results are in accordance with the results after Bonferroni corrections; only the angry mouth seemed to trigger longer fixations than the happy mouth (*p* = .03).

**Table 1:** Results in the baseline condition of the visual test phase analyses.

		Both faces	Angry face	Happy face	Value test	P value
		mean ± s.e.m	mean ± s.e.m	mean ± s.e.m		
		%	%	%		
**Neutral voice**	**Looking time at faces**	6.75 ± 0.53	3.75 ± 0.29	3.00 ± 0.23	F(1, 23) = 3.135	.09 NS
		68%	56%	44%		
	**Peak looks at faces**	691 ± 81	389 ± 51	301 ± 30	F(1, 23) = 2.02	.168 NS
	**Looking time at AOIs:**					
	**Mouth**	1.51 ± 0.36	0.94 ± 0.19	0.57 ± 0.17	F(1, 23) = 2.89	.103 NS
		22%	62%	38%		
	**Eyes**	1.43 ± 0.24	0.74 ± 0.12	0.69 ± 0.12	F(1, 23) = 0.15	.701 NS
		21%	52%	48%		
	**Peak looks at AOIs:**					
	**Mouth**	800 ± 186	523 ± 125	277 ± 186	F(1, 23) = 5.34	.014*
	**Eyes**	653 ± 125	317 ± 57	336 ± 68	F(1, 23) = 5.34	.689 NS
	**First fixations at faces** Number; % (Ntot = 48)	46; 96%	121; 46%	25425; 4%	t(47) = 0.58	.56 NS

Infants’ mean ± standard error and percentage looking time (s) and mean ± standard error of peak looks (ms) at faces and to AOIs and number and percentage of first fixations for the happy or angry face after the neutral voice.

*p < .05,

NS = Non Significant result.

### Preliminary analyses about the gender effect on looking times

A 2 (emotional voice familiarization condition: angry or happy) x 2 (gender: male or female) x 2 (emotional face: happy or angry) ANOVA was performed on the looking times with the voice conditions and emotional faces as a within-subjects factor and gender between-subject factors. The gender effect was not significant *(F*(1, 22) = .36, *p* = .56, *η* = .02) and did not interact with other factors (all p >.05).

A 2 (emotional voice familiarization condition) x 2 (gender) x 2 (emotional face) x 2 (AOIs: mouth or eyes) ANOVA was performed on the looking times, with the emotional voice conditions, AOIs, and the emotional faces as a within-subjects factor and gender as a between-subjects factor. The gender effect was not significant *(F*(1, 22) = .47, *p* = .50, *η* = .02) and did not interact with other factors (all p >.05). Consequently, results were further collapsed across gender.

### Main analyses: Looking times, first fixations and peak look at whole faces and AOIs

Table 2 presents the results of the visual test phase for the looking time at faces, the AOIs, the visual preferences of the infants, and their first fixations for the happy or angry face presented after the emotional voices (angry or happy).

**Table 2:** Results of the visual test phase analyses.

		Both faces	Angry face	Happy face	Value test	P value
		mean ± s.e.m	mean ± s.e.m	mean ± s.e.m		
		%	%	%		
**Happy voice**	**Looking time at faces**	5.33 ± 0.52	3.04 ± 0.24	2.29 ± 0.28	F(1, 23) = 4.85	.037*
		53%	56%	44%		
	**Peak looks at faces**	796 ± 140	446 ± 79	350 ± 61	F(1,23) = 1.30	.265 NS
	**Looking time at AOIs:**					
	**Mouth**	1.21 ± 0.31	0.83 ± 0.20	0.38 ± 0.11	F(1, 23) = 8.32	.008**
		12%	69%	31%		
	**Eyes**	0.99 ± 0.25	0.44 ± 0.11	0.55 ± 0.13	F(1, 23) = 0.54	.470 NS
		9%	45%	55%		
	**Peak looks at AOIs:**					
	**Mouth**	589 ± 114	347 ± 51	242 ± 64	F(1,23) = 2.43	132 NS
	**Eyes**	546 ± 102	243 ± 37	303 ± 65	F(1,23) = 1.09	.307 NS
	**First fixations at faces** (Ntot = 48)	44; 92%	29; 66%	15; 34%	t(47) = 2.19	.033*
**Angry voice**	**Looking time at faces**	4.91 ± 0.53	2.48 ± 0.24	2.42 ± 0.29	F(1, 23) = 0.04	.843 NS
		49%	56%	44%		
	**Peak looks at faces**	667 ± 101	313 ± 38	354 ± 63	F(1,23) = 0.36	.553 NS
	**Looking time at AOIs:**					
	**Mouth**	1.19 ± 0.29	0.72 ± 0.13	0.47 ± 0.16	F(1, 23) = 2.24	.148 NS
		12%	60%	40%		
	**Eyes**	0.44 ± 0.10	0.46 ± 0.09	0.69 ± 0.12	F(1, 23) = 0.04	.845 NS
		10%	49%	51%		
	**Peak looks at AOIs:**					
	**Mouth**	740 ± 182	429 ± 74	311 ± 109	F(1,23) = 1.21	.285 NS
	**Eyes**	492 ± 99	214 ± 46	278 ± 53	F(1,23) = 1.06	.313 NS
	**First fixations at faces** Number; % (Ntot = 48)	44; 92%	17; 36%	27; 64%	t(47) = 1.85	.069 NS

Infants’ mean ± standard error and percentage looking time (s) and mean ± standard error of peak looks (ms) at faces and to AOIs and number and percentage of first fixations for the happy or the angry face after the happy or angry voice.

*p < .05,

**p < .01,

NS = Non Significant result.

**Fig 5:**
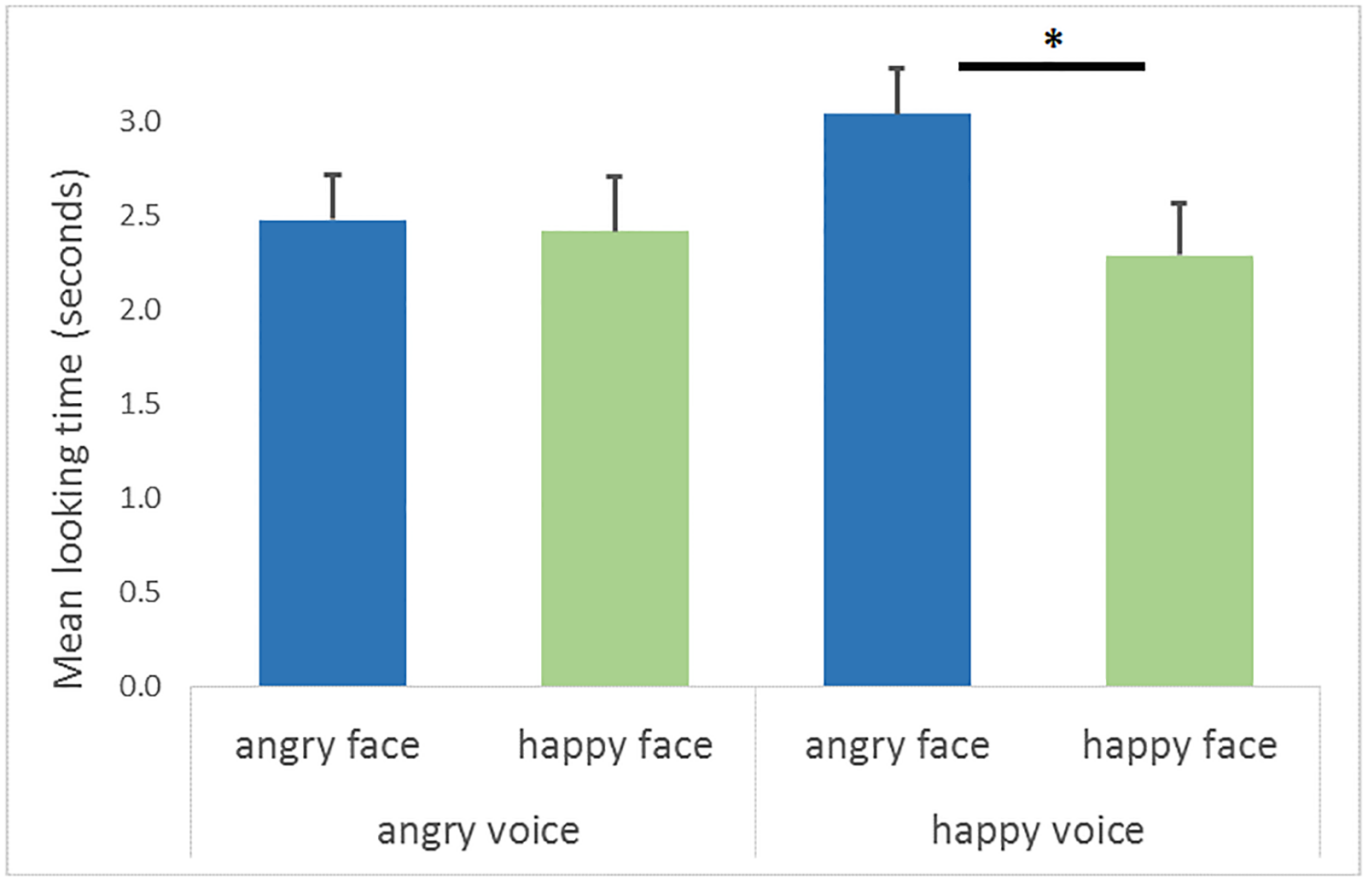
Looking time at happy or angry faces. Infants’ mean looking time (s) in function of voices (angry or happy) and emotional faces (angry: blue or happy: green). After hearing a happy voice, infants look longer at the angry face than the happy face (*F*(1, 23) = 4.85, *p* < .05). The vertical bars represent positive standard errors (s.e.m.),*p < .05.

A 2 (emotional voice familiarization condition: angry or happy) x 2 (emotional face: happy or angry, Fig 5) ANOVA was performed on the looking times, with the voice conditions and emotional face as a within-subjects factor.

The emotional voice familiarization condition was not significant (*F*(1, 23) = 1.51, *p* = .23, *η* = .06). The effect of the emotional face was significant (*F*(1, 23) = 7.42, *p* < .05, *η* = .244), with a clear visual preference for the angry face (mean ± s.e.m.; seconds, 2.76 ± 0.19 s.) compared to the happy face (2.35 ± 0.22 s.) presented. The interaction between the emotional voice familiarization condition and the emotional face was not significant (*F*(1, 23) = 1.43, *p* = .24, *η* = .058). Nevertheless, according to Iacobucci [45], it is possible to examine the effect of a non-significant interaction given certain conditions. He stated that if a simple effect is significant, we can explore its effect on the second, non-significant one. Under these circumstances, we can explore our a priori hypotheses. Therefore, pre-planned comparisons show that infants looked at the happy and the angry face equally after hearing the angry voice (*F*(1, 23) = .04, *p* = .843). By contrast, infants looked longer at the angry face than the happy face after hearing the happy voice, (*F*(1, 23) = 4.85, *p* < .05) (Fig 5). In sum, the looking time for the happy face is not affected by either emotional voice. However, the looking time for the angry face increases after hearing the happy voice.

**Fig 6:**
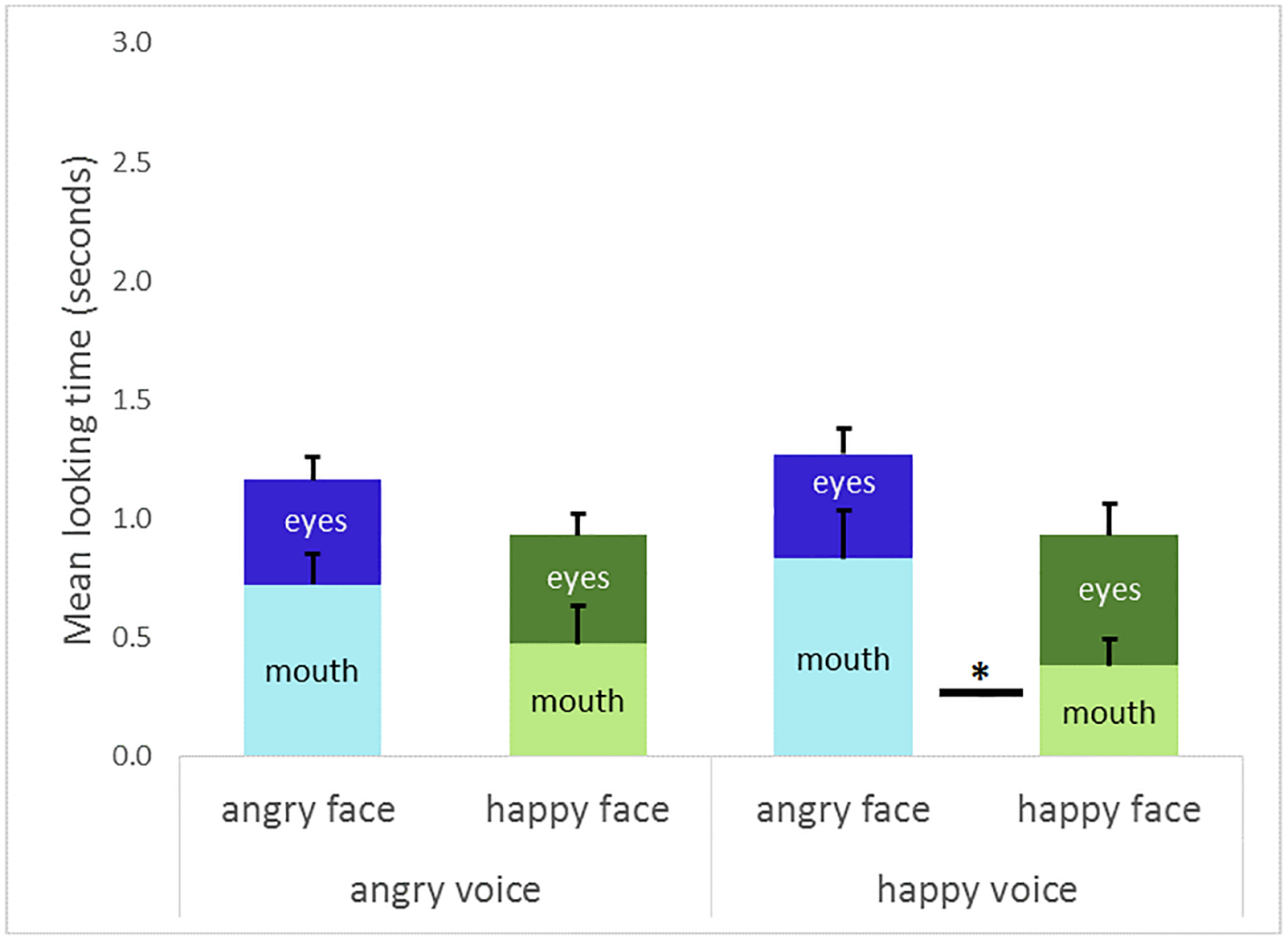
Looking times at happy or angry AOIs (mouth or eyes). Infants’ mean looking time (s) in function of the emotional voices (happy or angry) and emotional AOIs (happy: green or angry: blue). The angry mouth is looked at longer than the happy mouth *F*(1, 23) = 12.39, *p* < .01. After the happy voice, the angry mouth is looked at longer than the happy mouth (*F*(1, 23) = 8.32, *p* < .01). The vertical bars represent positive standard errors (s.e.m.), **p < .01.

A 2 (emotional voice familiarization condition) x 2 (emotional face) x 2 (AOIs: mouth or eyes, Fig 6) ANOVA was performed on the looking times, with the emotional voice conditions, AOI, and the emotional faces as within-subjects factors. Fig 6 presents the means and standard errors of looking times of the visual test phase for the AOIs (mouth and eyes) in function of the emotional faces (angry or happy) after each emotional voice familiarization condition (angry or happy).

The effect of AOIs was not significant (*F*(1,23) = .56, *p* = .46 *η* = .024). Infants seem to have looked at the mouth (mean ± s.e.m.; seconds, 0.60 ± 0.11 s.) and eye (0.47 ± 0.08 s.) areas for the same amount of time. The effect of the emotional voice familiarization condition was not significant (*F*(1, 22) = .25, *p* = .62, *η* = .011). The main effect of the emotional face was significant (*F*(1, 23) = 6.76, *p* < .05, *η* = .227). Infants also seem to have looked longer at the AOIs of the faces that expressed anger (0.61 ± 0.06 s.) than the AOIs of the faces that expressed happiness (0.46 ± 0.05 s.).

The interaction between the emotional voice familiarization condition and the AOIs was not significant (*F*(1, 23) = .08, *p* = .79, *η* = .003). The interaction between the AOIs and the emotional face was significant (*F*(1, 23) = 12.29, *p* < .01, *η* = .348). The results revealed that infants looked longer at the angry AOIs (0.60 ± 0.12 s.) than the happy ones (0.47 ± 0.08 s.). Pre-planned comparisons showed that infants looked longer at the mouth of the angry face (0.77 ± 0.14 s.) compared to the mouth of the happy face (0.42 ± 0.11 s.) (*F*(1, 23) = 12.39, *p* < .01), but no differences were shown between the happy or angry eyes (*F*(1, 23) = 1.32, *p* = .26).

The three-way interaction between the emotional voice familiarization condition, the AOIs, and the emotional face was not significant (*F*(1, 23) = .76, *p* = .39, *η* = .032). However, pre-planned comparisons showed that after the presentation of the happy voice, infants looked longer at the mouth of the angry face (0.83 ± 0.20 s.) than that of the happy faces (0.38 ± 0.11 s.) (*F*(1, 23) = 8.32, *p* < .01). There is no difference in looking time between the two emotional mouths (happy and angry) after the angry voice (*F*(1, 23) = .2.24, *p* = .148), no difference in looking time between the emotional eyes after the angry voice (*F*(1, 23) = .54, *p* = .47) or even the happy voice (*F*(1, 23) = .04, *p* = .845). In sum, the looking time for the happy AOIs is not affected by either emotional voice. However, infants increased their looking time for the angry AOIs after hearing a happy voice, particularly for the mouth area (Table 2).

Furthermore, a single Student’s T-test analysis was conducted to examine the first fixation on either emotional face (angry or happy) in function of the emotional voice (angry and happy) presented beforehand. After hearing a happy voice, infants directed their first fixation more often toward the angry face (66%) than the happy face (34%) (t(47) = 2.19, p < .05). By contrast, the angry voice had a tendency effect on the first fixations, as the infants tended to direct their first fixation more often to the happy face (64%) than the angry face (36%) (t(47) = 1.85, p = .069). As previously stated, after being exposed to the neutral voice, infants directed their first fixation equally, to both faces (Table 1).

Moreover, a 2 (emotional voice) x 2 (emotional face) ANOVA was performed on the peak looks. We found no significant difference concerning the peak looks at the emotional face (*F*(1, 23) = 0.296, *p* = .59, η = 0.01), in function of the voice (*F*(1, 23) = 1.214, *p* = .28, η = 0.05) or interaction between emotional faces and voices *(F*(1, 23) = 1.42, *p* = .24, η = 0.06).

A 2 (emotional voice) x 2 (emotional face) x 2 (AOIs: mouth or eyes) ANOVA was performed on the peak looks. We only found a significant interaction between AOI and emotional faces (*F*(1, 23) = 4.45, *p* < .05, η = 0.16). After Bonferroni corrections, no significant difference was found, only a tendency of longer peak looks at the angry mouth (388.04 ± 51.49 ms) compared to the angry eyes (228.47 ± 34.94 ms), suggesting that with the angry face, the mouth involves the longest peak looks.

### Complementary analyses of the voice effect: Neutral vs emotional voices

After the 3 voices (neutral, happy and angry), the results on looking time at faces suggested that the effect of the emotional voice was significant (*F*(2, 46) = 11.05, *p* < .001, *η* = .32). Indeed, after Bonferroni corrections, in the neutral voice trials (first 2 trials), the faces are looked at longer than after the emotional voices (angry voice: p = .002; happy voice: p = .008).

The emotional face condition was significant (*F*(1, 23) = 13.026, *p* < .01, *η* = .362), with longer looking time at the angry face (3.09 ± 0.17 s.) compared to the happy face (2.57 ± 0.17 s.). The interaction between the emotional voice familiarization condition and the emotional faces was not significant (*F*(1, 23) = 0.96, *p* = .39, *η* = .04).

Results of the infants’ looking time at the AOIs (mouth and eyes) showed that the emotional voice condition was significant (*F*(2, 46) = 7.823, *p* < .001, *η* = .25). After the neutral voices, the faces are looked at longer than after the emotional voices. The main effect of the emotional faces was significant (*F*(1, 23) = 8.44, *p* < .01, *η* = .268). Furthermore, the interaction between AOIs and emotional faces was also significant (*F*(1, 23) = 10.06, *p* < .01, *η* = .304). After Bonferroni corrections, the angry mouths were looked at longer than the happy mouths (*p* = .002), the angry eyes (*p* = .014) or the happy eyes (*p* = .03). The three-way interaction between the emotional voice familiarization condition, the AOIs, and the emotional faces was not significant (*F*(1, 23) = .518, *p* = .59, *η* = .022.

Concerning the first fixations, as previously stated after being exposed to the neutral voice, infants directed their first fixations equally to both faces (t(47) = 0.58, p = .56) while after the angry voice infants had a tendency to direct more their first fixations at the happy face (t(47) = 2.19, p < .05) and after the happy voice they directed their first fixation more to the angry face (t(47) = 1.85, p = .069).

Finally, with the 3 voices, no significant difference was found regarding the peak looks at the faces. The peak looks at the AOI reveled a tendency effect in emotional face condition (*F*(1, 23) = 3.73, *p* = .065, *η* = .139, suggesting that angry AOIs trigger longer peak looks than happy AOIs. Moreover, the interaction between AOIs and emotional faces was significant, indicating after Bonferroni correction, that angry mouths trigger longer peak looks than happy mouths *p* = .01, angry eyes *p* = .005 or happy eyes tend to *p* = .068.

## Discussion

The main goal of this study was to determine if 6-month-old infants are able to extract amodal components in emotional facial expressions (happiness or anger) through a cross-modal transfer paradigm—from auditory to visual modalities. The results showed that this ability differs depending on the type of emotional faces and voices presented (happy or angry). Specific facial areas, such as the eyes and mouth, can also influence their preference.

The first basic result observed in the baseline condition (neutral voice) was the absence of a significant spontaneous visual preference between the two emotional faces and specific areas by 6-month-old infants. This absence of visual preference for one or the other facial expression was expected with the neutral voice. Indeed, the angry and the happy faces are both novel in regards to the neutral voice so both faces are equally looked at. This result also confirms that the preference for happiness seems to decline with age, especially in a paradigm involving two alternative auditory and visual sequences: 20 sec (no faces)—10 sec (two faces) respectively. It may be that these conditions eliminate a spontaneous visual preference for happiness. An additional complementary explanation would be the emergence of fear sensitivity between the age of 5 and 7 months [46–48]. For example, an attentional and arousal response has been observed in 6–7 month-old children in response to audiovisual stimuli of infants crying using pupillometric measures [49]. In adults, this may parallel the specific engagement of threat processing pathways (amygdala) by auditory screams (e.g. [50]). This supports the idea of a potential developmental shift in emotional processing at 6- to 7-months [4]. The developmental trajectory of the sensitivity to each emotional expression (joy, anger, fear, etc.) from birth to 6-7-months and its underlying mechanisms remain under debate [3]. In any case, this absence of preferences after the neutral voice allows us to better explore whether emotional voices can influence the visual preferences for an emotional face.

The second result showed that visual preference (looking times and peak looks) and the first look changed depending on the hearing familiarization phase. After listening to the angry voice, differences in visual preferences (whole face and specific areas) or first fixations, were not observed between the two faces. By contrast, after listening to the happy voice, infants looked longer at the angry (novel) face than at the happy (familiar) face. These results are consistent with the previous research, which observes that happy is the emotion that is recognized most precociously [3].

Studies on AOIs in emotional recognition have suggested that AOIs observed can predict emotions perceived by the subject [29]. In our study we observed that infants looked longer at the mouth area of an angry face compared to the mouth area of a happy one. However, they looked at the eyes similarly for both faces. This result is in agreement with the preliminary results presented by Hoehl [51] which stated that the mouth area of an angry face is preferred by 7 month-old infants.

However, these results can be influenced by the saliency of the mouth area in the angry face we used, which was fully open. Therefore, the mouth of the angry expression, which was looked at longer and triggered the longest peak looks independently of the voice presented, could have drawn more attention than the mouth of the happy face. Moreover, Caron and collaborators [34] have demonstrated that 5-month-old infants are influenced by the presence of teeth when interpreting facial emotions. However, it can be noted that with the faces we used in our experiment, both faces presented visible teeth, even though the teeth were more salient in the angry faces. According to Oster & Evy (1980) quoted by Oster [52], 4-months-old infants differentiate between happy and sad faces if teeth are visible, but are unable to distinguish between these two emotions if teeth are not visible for the happy face. In agreement with these authors, we hypothesize that babies evaluate emotions more from the pictorial elements of faces. Nevertheless, it is after listening to a happy voice that the difference of looking time between the angry and happy mouth is present, indicating that it’s the novel incongruent mouth drives attention more.

In the same way, our results showed that after listening to a happy voice, the first fixations of infants were more drawn to the angry face (novel) than the happy (familiar) face. These results are consistent with adult studies showing that the visual processing of the face is extremely fast in adults (fewer than 150 ms) [53], suggesting that the first fixation could be oriented by the emotional nature of stimuli, and is done so from an early age.

The existence of a cross-modal transfer (from audio to visual modalities) after the happy voice in 6-month-old infants may be related to the greater familiarity to happiness compared to anger. Additionally, vocal stimuli of anger could be considered less ecological than those of happiness. Indeed, it is rare to repeat angry vocalizations endlessly compared to a happy voice or a real laugh. If we can say that the vocal stimulus is much more familiar (happy in the present experiment), babies should have a preference for novelty and prefer to explore the incongruous facial expression (anger). If a voice is less familiar (angry in the present experiment), most babies will not express a preference for one or the other facial expression (anger or happiness). To verify this interpretation, it would be wise to study whether a child could link a familiar sound to its respective object, thus influencing a visual preference.

Finally, it is noteworthy that in all the studies mentioned above, most researchers are not interested in the "gender effect" of babies. Concerning our study, we found no differences between the male and female infants in accordance with Kuchuk et al. and Labarbera et al. [38,39].

In conclusion, these results revealed that the angry mouth area drives the most attention and that a cross-modal transfer (from auditory to visual modalities) is possible for 6-month-old infants only after the presentation of a happy voice, suggesting that they recognize this emotion amodally.

## Supporting information

S1 Dataset LookingTimes. (XLSX)

S2 Dataset Peaklooks_Max fixation. (XLSX)

S3 Dataset FirstFixation. (XLSX)
